# The Role of Whole Genome Sequencing in the Surveillance of Antimicrobial Resistant *Enterococcus* spp.: A Scoping Review

**DOI:** 10.3389/fpubh.2021.599285

**Published:** 2021-06-10

**Authors:** Lindsay A. Rogers, Kayla Strong, Susan C. Cork, Tim A. McAllister, Karen Liljebjelke, Rahat Zaheer, Sylvia L. Checkley

**Affiliations:** ^1^Department of Ecosystem and Public Health, Faculty of Veterinary Medicine, University of Calgary, Calgary, AB, Canada; ^2^Lethbridge Research and Development Centre, Agriculture and Agri-Food Canada, Lethbridge, AB, Canada

**Keywords:** *Enterococcus*, whole genome sequencing, scoping review, antimicrobial resistance, surveillance, One Health, nosocomial infection

## Abstract

*Enterococcus* spp. have arisen as important nosocomial pathogens and are ubiquitous in the gastrointestinal tracts of animals and the environment. They carry many intrinsic and acquired antimicrobial resistance genes. Because of this, surveillance of *Enterococcus* spp. has become important with whole genome sequencing emerging as the preferred method for the characterization of enterococci. A scoping review was designed to determine how the use of whole genome sequencing in the surveillance of *Enterococcus* spp. adds to our knowledge of antimicrobial resistance in *Enterococcus* spp. Scoping review design was guided by the PRISMA extension and checklist and JBI Reviewer's Guide for scoping reviews. A total of 72 articles were included in the review. Of the 72 articles included, 48.6% did not state an association with a surveillance program and 87.5% of articles identified *Enterococcus faecium*. The majority of articles included isolates from human clinical or screening samples. Significant findings from the articles included novel sequence types, the increasing prevalence of vancomycin-resistant enterococci in hospitals, and the importance of surveillance or screening for enterococci. The ability of enterococci to adapt and persist within a wide range of environments was also a key finding. These studies emphasize the importance of ongoing surveillance of enterococci from a One Health perspective. More studies are needed to compare the whole genome sequences of human enterococcal isolates to those from food animals, food products, the environment, and companion animals.

## Introduction

A variety of *Enterococcus* spp. are commensals within the gastrointestinal tract (GIT) of humans and animals, while others exist within the broader environment; some enterococcal species have also emerged as important human pathogens, especially in nosocomial infections (1). Two enterococcal species, *Enterococcus faecium* and *Enterococcus faecalis*, are most commonly implicated in human disease (2). These species, in particular, can acquire antimicrobial resistance (AMR) and harbor virulence genes that give them advantages as opportunistic pathogens (3). Their acquisition of antimicrobial resistance genes (ARGs) can be chromosomally- and plasmid- mediated, arising from selection pressure through antimicrobial use and the transfer of ARGs on mobile genetic elements (MGEs) such as plasmids and transposons (4). Of particular concern is the rising emergence of vancomycin-resistant enterococci (VRE) (4). Acquired vancomycin resistance is mediated by various gene clusters termed VanA/B/D/E/G/L/M/N (4, 5). Each gene cluster codes for a different resistance mechanism. VanA and VanB are the most common clusters seen, are often hospital-acquired, and can be plasmid- or chromosomally- mediated (5–7). Two species, *Enterococcus gallinarum* and *Enterococcus casseliflavus*, have intrinsic vancomycin resistance that is chromosomally-mediated by the VanC gene cluster (4, 5).

These two species, along with others such as *Enterococcus villorum, Enterococcus thailandicus, Enterococcus durans*, and *Enterococcus hirae*, are more typical of animal- and environmentally- adapted species (8, 9). Their genomes reflect adaptations to specific niches; for example, clusters of orthologous groups (COGs) have been found for ethanolamine utilization as a carbon source in environmental species that are not found in *E. faecium* (8).

Other antimicrobials can co-select for vancomycin-resistance genes if there are multiple ARGs on a single mobile genetic element. This means that the use of other antimicrobials can lead to the acquisition of vancomycin-resistance genes, even if vancomycin is not used (10). Thus, the VRE that arise in this manner are also multi-drug resistant (MDR) or multi-class resistant (7) referring to organisms that have acquired resistance to two or more antimicrobials or those organisms that have acquired resistance to two or more classes of antimicrobials, respectively.

As AMR pathogens have emerged, the use of antimicrobials for prophylaxis and metaphylaxis in food producing animals has come under scrutiny for its potential to apply a selective pressure that contributes to the dissemination of AMR and MDR enterococci (7, 11). Perhaps the most classic example is in poultry and swine where the previous use of a vancomycin-related glycopeptide, avoparcin, as a growth promoter was associated with carriage of vancomycin-resistant *E. faecium* (VREfm) in treated herds or flocks through cross-resistance (7, 12). The occurrence of VREfm in food animals decreased after the avoparcin use in animals was banned (12); however, prolonged persistence of specific VREfm clusters in agricultural settings (e.g., VanA gene cluster on the Tn*1546* transposon) were observed possibly due to co-selection for VRE through continued use of other antimicrobials, such as macrolide use in swine (7, 12, 13). Retrospective molecular and genetic studies have demonstrated that the VREfm isolated from hospital and agricultural setting are usually separate sequence types (7, 12, 13). In contrast, the same sequence types of vancomycin-resistant *E. faecalis* can be isolated from hospital settings and from farm animals (7, 12, 14). In human medicine, enterococci are often opportunistic pathogens that acquire resistance and arise in immunocompromised individuals in hospital settings. Often these patients have been treated with multiple classes of antimicrobials in an effort to control difficult to treat infections (6). These antimicrobials may include those deemed critically important for use in human medicine by WHO (10), leading to enterococci resistant to these antimicrobials circulating in the human population (10).

Due to the importance of *Enterococcus* spp. as potential human pathogens, their ability to easily acquire ARGs, and their ubiquitous nature in the GIT and the broader environment, many countries have added enterococcal species to their list of pathogens under surveillance. Their role as a GIT commensal also make enterococci useful as fecal indicator bacteria (15). Surveillance for *Enterococcus* spp. and the other so-called ESKAPE pathogens (ESKAPE is an acronym for the following six nosocomial pathogens: *Enterococcus faecium, Staphylococcus aureus, Klebsiella pneumoniae, Acinetobacter baumannii, Pseudomonas aeruginosa*, and *Enterobacter* spp.) (16) is done on a national level in several countries. These include programs such as the Danish Integrated Antimicrobial Resistance Monitoring and Research Program (DANMAP) (17), USA's National Antimicrobial Resistance Monitoring System (NARMS) (18), the Canadian Integrated Program for Antimicrobial Resistance Surveillance (CIPARS) (19), and the Colombian Integrated Program for Antimicrobial Resistance Surveillance (COIPARS) (15). Many hospitals and laboratories also have surveillance systems in place for specific organisms independent of national surveillance programs. Examples include JMI Laboratories' SENTRY Antimicrobial Surveillance Program and the University of Pittsburgh Medical Center-Presbyterian Hospital's Enhanced Detection System for Hospital-Acquired Transmission (UPMC EDS-HAT) (6, 20, 21). The European Antimicrobial Resistance Surveillance Network (EARS-Net) is a large surveillance program based on clinical antimicrobial resistance data from laboratories across Europe (22). The pharmaceutical industry also runs some important post-marketing surveillance programs to comply with licensing requirements of new antimicrobials, looking at potency and spectrum. Examples of these programs are the Zyvox Annual Appraisal of Potency and Spectrum (ZAAPS) and the Linezolid Experience and Accurate Determination of Resistance (LEADER) (23). Integrated surveillance programs survey and address AMR in humans, animals, and the environment from a One Health perspective, emphasizing the interfaces within the system. These programs collect samples and process isolates from various sources, including animal fecal samples, human screening samples, retail meats, wastewater, surface water, groundwater and soils (15). Human screening samples include those from hospital surveillance programs of in-patients and samples submitted to laboratories performing surveillance (6, 24–26). The data generated from the wide range of samples can be integrated with antimicrobial use data allowing for the monitoring of changes in antimicrobial resistance found in bacteria important to public and animal health (15). Information gleaned from surveillance can then inform policy and risk mitigation strategies to combat increasing AMR and protect antimicrobials important to human health (15). For example, surveillance of VRE through DANMAP allowed for the detection of VRE strains in broiler chickens and human isolates connected to the use of avoparcin for growth promotion in broilers. This detection led to the ban of avoparcin use in food animal production (7, 15).

Early surveillance was primarily based on traditional microbiology to determine phenotypic antimicrobial susceptibility profiles, molecular genomics methods such as polymerase chain reactions (PCR) to assess for the presence of resistance genes, and pulsed-field gel electrophoresis (PFGE) for DNA fingerprinting Multi-locus sequence typing (MLST) arose more recently to better assess genetic relationships among isolates (7, 27). The use of these technologies allowed for the phylogenetic study of sequence types, epidemiologic investigation and determination of the presence of specific ARGs. However, the development of whole genome sequencing (WGS) has provided a more in-depth and detailed analysis of enterococcal ARGs, phylogenetics, and virulence (7, 27). As whole genome sequencing has become more widely available and less expensive, many archived isolate collections are being reanalyzed and their genomes compared with new isolates (13, 28). Following WGS, it has become possible to utilize new sequence typing methods for enterococci, such as core-genome multi-locus sequence typing (cgMLST), allowing for better analysis of isolate relatedness across sample sources (28). WGS also allows for the identification of emerging strains, analysis of outbreaks, and the characterization of resistance and virulence genes and their locations and context in the bacterial genome. Due to these advantages many surveillance research groups have been transitioning to WGS-based approaches of isolate characterization. Sequencing compliments traditional microbiology approaches and offers a reliable method of characterizing ARGs and sequence types (27).

With the increasing popularity of WGS for bacterial pathogen surveillance, it is now imperative to review the progress that has been made toward surveillance methods and identify gaps in our surveillance knowledge. We have undertaken this scoping review to investigate and summarize the extent to which whole genome sequencing in surveillance studies has advanced our understanding of AMR in *Enterococcus* spp.

## Methods

To investigate our research question, a scoping review was designed following the PRISMA-ScR extension for scoping reviews (29) and the guidelines laid out by the JBI Reviewer's Manual (30). This protocol was not registered with an online registration platform.

### Search Terms and Strategy

The Population, Concept, Context (PCC) framework (30) was used to develop the research question and search strategy. A search strategy was developed to return a broad range of studies that fit within the following population, concept, and context:

- Population: *Enterococcus* spp. that underwent whole genome sequencing
- Context: *Enterococcus* spp. isolates derived from surveillance-type studies (as described below)
- Concept: use of whole genome sequencing to better understand antimicrobial resistance.

After consultation with a librarian, three separate databases were selected for searching: PubMed (NCBI)^1^, Web of Science^2^, and CAB Abstracts^3^ All databases within Web of Science were included in order to include both the Web of Science Core Collection and BIOSIS. Searches were performed by a single reviewer (LR) on November 17, 2019, and email search alerts from each database were implemented to inform the reviewer of any new articles. New articles from alerts up to and including December 31, 2020 were included. The same search terms were used for all three databases, except for differences due to specific database formatting.The search terms and resulting number of articles for one database (Web of Science) are described in Table 1. To capture relevant gray literature, a manual search of the reference lists of included articles was completed during data extraction. Gray literature is information produced and distributed outside of academic publications such as government reports. The gray literature underwent the same screening process; however, the full text was read for screening if no abstract was available. The web application, Rayyan, was used for the organization of articles during the screening process (31).

**Table 1:** Search results table from Web of Science database search with search terms and resulting number of articles.

Set^*^	**Results^**¥**^**	**Search terms^**‡**^**
4	283	#3 AND #2 AND #1 *Databases= WOS, BIOSIS, KJD, MEDLINE, RSCI, SCIELO Timespan=2002-2020* *Search language=English*
3	3,139,503	**TOPIC:** (survey) *OR* **TOPIC:** (surveillance) *OR* **TOPIC:** (epidemiolog^*^) *Databases= WOS, BIOSIS, KJD, MEDLINE, RSCI, SCIELO Timespan=2002-2020* *Search language=English*
2	485,212	**TOPIC:** (whole genome sequenc^*^) *OR* **TOPIC:** (WGS) *OR* **TOPIC:** (next generation sequenc^*^) *OR* **TOPIC:** (NGS) *OR* **TOPIC:** (genomic^*^) *Databases= WOS, BIOSIS, KJD, MEDLINE, RSCI, SCIELO Timespan=2002-2020* *Search language=English*
1	46,024	**TOPIC:** (enterococc^*^) *OR* **TOPIC:** (enterococcus) *Databases= WOS, BIOSIS, KJD, MEDLINE, RSCI, SCIELO Timespan=2002-2020* *Search language=English*

* *Set: Number assigned by Web of Science database to search term*.

¥ *Results: Number of articles in Web of Science database matching search terms for each set*.

‡ *Search terms: Terms used in each set for the search. The numbers given in Set 4 represent the combination of other sets used for the search*.

### Article Screening and Selection

Only journal articles and abstracts in the English language, published after 2002 were eligible for screening. The entire genome of *E. faecium* was first sequenced in 2000 (32); however, the assembly was not completed until 2012 (33, 34). The earliest available genome sequence of an *Enterococcus* sp. from the NCBI database is from 2002 (35). Given this information, 2002 was considered the earliest year that a publication would contain the information relevant to this scoping review. Any publication that was not an article or abstract (e.g., textbook, poster, or conference presentation) was excluded. Relevant gray literature articles were included and searched for, as described above. All screening was done independently by two reviewers (LR and KS). Any discrepancies were resolved in discussion with two other reviewers (SLC and SCC).

#### Title Screening

The article titles were screened initially and any article that was clearly about bacteria other than *Enterococcus* spp. was excluded. These articles needed to explicitly include the name of bacteria other than *Enterococcus* spp. in the title and not include terms relating to the taxonomy of enterococci. All articles that did not meet this exclusion criterion were included for the next screening step. The title screening was intentionally left broad to maximize the number of articles included.

#### Abstract Screening

Two screening steps were applied to the included abstracts. The first abstract screening step was performed to exclude any articles that did not include whole genome sequencing and antimicrobial resistance of *Enterococcus* spp. The abstract had to include all three pieces of information (i.e., WGS, AMR, and *Enterococcus*) in the abstract text. This step was also intentionally left broad and any articles that mentioned sequencing without providing information about whether or not the whole genome was sequenced were included to be screened based on methodology (described below). Following this, the abstracts were screened a second time using the following question, “did all or a portion of the *Enterococcus* spp. isolates in this study result from surveillance or screening for enterococci?” The following criteria for surveillance or screening were used:

- Isolates were from a collection maintained by a surveillance group (a surveillance group is defined as an organization collecting and analyzing bacterial isolates for surveillance of those particular bacteria such as CIPARS, SENTRY, or DANMAP). OR,
- A statement was included that the isolates were collected for screening or surveillance purposes. OR,
- Isolates were collected for the sole purpose of genomic comparison. OR,
- The article was published in a journal which included “Surveillance” in the journal name.

Articles needed to meet one or more criteria. Articles that did not meet these criteria, such as those with only clinical isolates, were excluded. Articles that were unclear if they met the surveillance inclusion criteria through their abstract were screened based on methodology as described below.

#### Methods Screening

As stated previously, some abstracts did not contain enough detail to determine if they met the inclusion/exclusion criteria. These articles were further screened through the reading of their methods sections, following the same criteria as for abstract screening. Articles that did not meet the inclusion criteria during the methods review were excluded.

**Figure 1:**
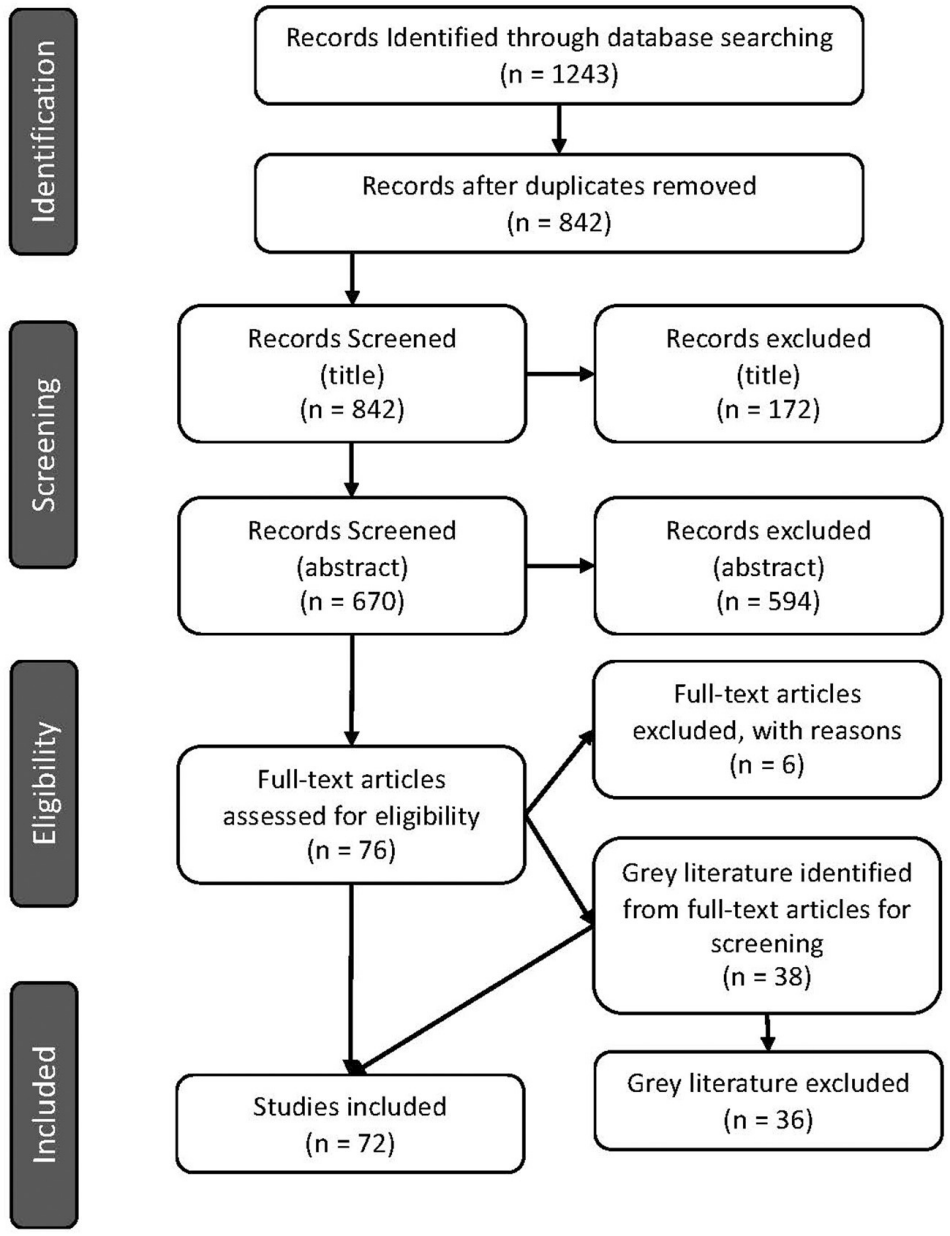
PRISMA flow diagram indicating number of articles screened and identified for inclusion in the scoping review (29).

The number of articles excluded at each screening step is displayed in Figure 1.

### Data Extraction and Charting

Data was extracted independently by two reviewers (LR and KS) to answer the research question. The chart was trialed with 15 articles to ensure the reviewers were extracting comparable information. The completed tables from data extraction were compared by one reviewer (LR), and any discrepancies in information were resolved in discussion between the two original reviewers (LR and KS). No critical appraisal of articles was performed and all articles were included regardless of study quality. The methodology of data extraction is outlined in Table 2.

**Table 2:** Data extraction chart with description and example article.

**Data**	**Description**	**Example**
**Article characteristics**		
Citation	Article reference using APA 6th style	Leong, K. W., Cooley, L. A., Anderson, T. L., Gautam, S. S., McEwan, B., Wells, A., Wilson, F., Hughson, L., and O'Toole, R. F. (2018). Emergence of vancomycin-resistant *Enterococcus faecium* at an Australian Hospital: a whole genome sequencing analysis. *Scientific Reports* 8:6274.
Article type	List the study design or type	Cross-sectional
Research group country	Name the country from where the research group originates	Australia
Surveillance group	List the surveillance group(s) performing the study and/or with the isolate collection (if applicable)	Tasmania Infection Prevention and Control Healthcare Associated Infection Surveillance Program
Funders	List the funding groups for the study	- Royal Hobart Hospital Research Foundation grant - Tasmanian Infection Prevention and Control Unit
**Introduction**		
Study objectives	Bullet point summary of stated objectives	- To better define hospital spread of VREfm at the RHH - Will correlate genomic information with epidemiologic data
**Methods**		
Study location	List where the samples are from or where the study was performed	Royal Hobart Hospital (RHH) in Tasmania
*Enterococcus* spp.	List the enterococcal species isolated and studied	*E. faecium*
VREfm specific study (yes/no)	Yes: the study is specifically studying VREfm No: the study is not specifically looking for VREfm (although it may be included in the results)	Yes
Sample sources	List the sources from where the isolates were obtained	- Human screening - Human clinical
AMS testing	List the methods used for antimicrobial susceptibility testing	Done in previous study—disc diffusion (EUCAST)
WGS Platform	List the WGS platform(s) used	Illumina MiSeq
Archive accession numbers provided (yes/no)	Yes: accession numbers provided No: accession numbers not provided	Yes
Bioinformatic tools	List any bioinformatics tools used according to their purpose	Alignment: Snippy Phylogeny (SNPs): RaxML Assembly: Velvet AMR: Resfinder Typing: MLST Tool
Other genomics	List any other genomic analyses performed	None
**Results**		
AMR phenotypes	List the antimicrobials to which resistance was found (percent or ratio of isolates in parentheses) Make note of any particular phenotypic patterns	- Vancomycin (100%)
Sequence types and/or clonal complexes	List any sequence types and/or clonal complexes found	ST796 (47/80) ST80 (16/80) ST1421 (10/80) ST203 (4/80) ST78 (1/80) ST192 (1/80) ST555 (1/80)
AMR genes	List of AMR genes found through WGS, according to sample source If >10 AMR genes were found, reference where in the article they may be found, rather than providing an exhaustive list	*vanA* *vanB*
Plasmids	List of any plasmids found	None described
Relatedness assessed	Brief description of the relatedness that was assessed	Study isolates to reference genome (SNPs) Comparison of genomic to epidemiologic data
**Discussion**		
Addition to AMR knowledge	Summary of addition to knowledge about AMR in *Enterococcus*	- VREfm profile at RHH has shifted to ST796 and ST80 - Screening is important to detect isolates that may be involved in transmission - WGS is helpful for more accurate typing of *E. faecium* for better investigations

## Results

### Article Characteristics

**Figure 2:**
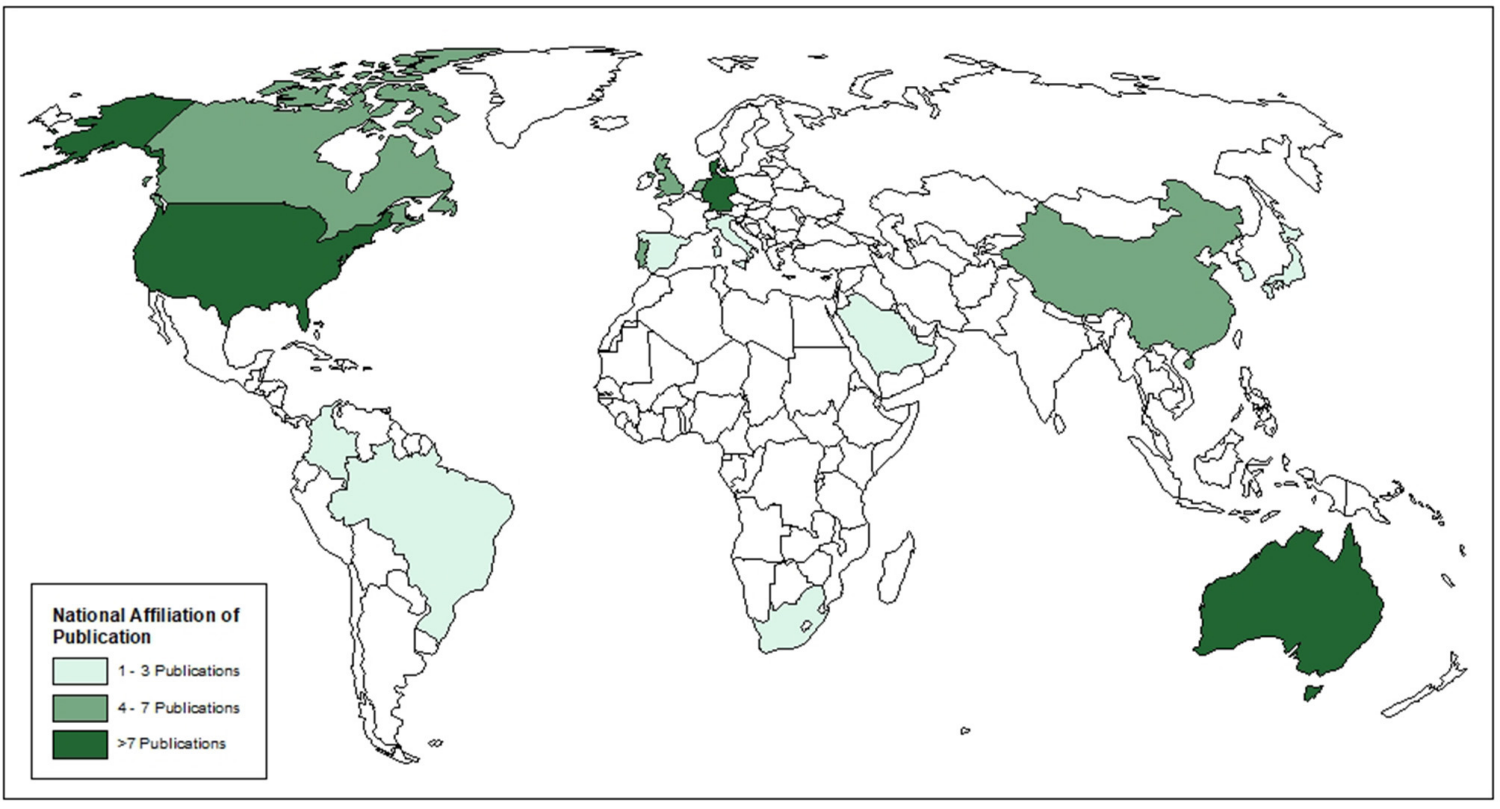
Country of affiliation of corresponding author for included articles.

Seventy-two articles were included after the full-text review (Figure 1). Of these, 70 were primary research articles and two were gray literature (government reports) (25, 36). All articles were published in 2015 or later. The corresponding authors were from seventeen (17) different countries with most from Australia (16.7%), Denmark (13.9%), and Germany (13.9%), followed by the USA (12.5%), then the UK (6.9%) and 5.6% from each of Canada, China, the Netherlands and Portugal. Corresponding authors were also from Brazil (4.2%), Italy (1.4%), Japan (1.4%), Colombia (1.4%), Spain (1.4%), South Africa (1.4%), South Korea (1.4%), and Saudi Arabia (1.4%) (Figure 2). Additional information is available in Supplementary Table 1.

Just under half of the studies were not associated with a specified surveillance group (48.6%). The remaining articles were associated with government funded programs (e.g., DANMAP, NARMS), within hospital screening or surveillance programs, or private/industry funded surveillance programs (Table 3 and Supplementary Table 1).

**Table 3:** Proportion of articles funded and/or performed by a specific surveillance group.

**Specified surveillance group**	**Proportion of articles (%)**	**Article citation**
None listed	48.6	(8, 9, 11, 24, 37–67)
Government funded program	30.6	(25, 27, 28, 36, 68–85)
Private/Industry funded program	4.2	(13, 26, 86)
Within hospital program	16.7	(6, 20, 87–96)

### Objectives of Included Articles

The study objectives could be broadly divided into five categories, as shown in Table 4. These include (i) epidemiology or prevalence studies, (ii) genetic and/or molecular characterization, (iii) comparative genomics, (iv) the description of a novel finding, and (v) the comparison of techniques or development of a new technique. The categories were formed by assessing keywords within the stated objectives. For example, objectives to “determine the prevalence of…” or “perform comparative genomics of…” would fit into the categories of epidemiology or prevalence studies and comparative genomics, respectively. The categories were further broken down into the sample source location, and whether or not the article was studying a specific resistance gene (e.g., *vanA*), AMR profile (e.g., VRE), or another specific category (e.g., another specific genetic element such as a transposon). Articles could fall into more than one type of objective but generally stayed within the same category as the source location. The majority of articles were an epidemiological or prevalence study (54.2%), most commonly conducted within a defined region. Often these studies included human clinical data and hospital isolates but were not specific to just one hospital. Only three studies compared techniques or developed new ones, and this was in conjunction with other objectives of the study. Details of the objectives for each study are presented in Table 4 and Supplementary Table 2.

**Table 4:** Categorization of stated objectives for articles included in scoping review and proportion of included articles in each category.

**Objective category and source**	**Article specific to a gene, AMR profile, or other genetic element?**	**Proportion of articles (%)**	**Article citation**
**Epidemiology and/or prevalence study (54.2%)**			
Hospital or clinical setting only	Yes	16.7	(6, 26, 46, 49, 62, 72, 73, 90, 92, 93, 95, 96)
	No	1.4	(64)
Regionally specific study (may include clinical setting)	Yes	20.8	(24, 27, 28, 37, 45, 47, 50, 63, 74, 75, 78–80, 82, 85)
	No	4.2	(25, 36, 65)
Outbreak study	Yes	6.9	(20, 51, 52, 87, 94)
	No	–	
Food animal related (including retail meats and animal samples)	Yes	2.8	(55, 67)
	No	–	
Other	Yes	–	
	No	1.4	(41)
**Comparative genomics (29.1%)**			
Regionally specific study (may include clinical setting)	Yes	8.3	(28, 56, 63, 75, 77, 83)
	No	1.4	(53)
Food animal related (including retail meats and animal samples)	Yes	–	
	No	6.9	(8, 13, 38, 68, 84)
Other (multiple sources)	Yes	2.8	(58, 59)
	No	9.7	(9, 38–41, 54, 57)
**Genetic and/or molecular characterization (23.5%)**			
Hospital or clinical setting only	Yes	8.3	(26, 48, 49, 60, 89, 93)
	No	–	
Regionally specific study (may include clinical setting)	Yes	6.9	(44, 66, 75, 81, 91)
	No	–	
Food animal related (including retail meats and animal samples)	Yes	6.9	(55, 61, 67, 69, 70)
	No	1.4	(54)
**Description of novel finding (14.0%)**			
Hospital or clinical setting only	Yes	2.8	(60, 86)
	No	–	
Regionally specific study (may include clinical setting)	Yes	4.2	(37, 74, 81)
	No	–	
Outbreak study	Yes	1.4	(88)
	No	–	
Food animal related (including retail meats and animal samples)	Yes	2.8	(11, 71)
	No	–	
Other	Yes	1.4	(43)
	No	1.4	(76)
**Comparison or development of technique(s) (5.6%)**			
Hospital or clinical setting only	Yes	2.8	(6, 92)
	No	–	
Regionally specific study (may include clinical setting)	Yes	1.4	(27)
	No	–	
Outbreak study	Yes	–	
	No	1.4	(42)

### Enterococcal Source and Species

Nine different source types were sampled for surveillance of *Enterococcus* spp. isolates, with the majority of articles using isolates from human clinical infections (47 articles, 65.3%). Just over half of the articles (38 articles, 52.8%) used isolates from human screening samples. For animal samples, 12 articles (16.7%) pulled isolates directly from food animals (fecal, cecal, or other samples), 12 articles (16.7%) had retail meat samples, and three articles (4.2%) included isolates from milk products. Isolates were also from environmental samples with eight articles (11.1%) including samples from the hospital environment and eight articles (11.1%) using wastewater samples. Five articles (6.9%) included sequences from GenBank to supplement their isolates from sample sources (Table 5). No studies sampled from companion animals or equids.

**Table 5:** Summary of enterococcal source and species from articles included in scoping review and proportion of included articles identifying each source or species.

**Enterococcal characteristics**	**Proportion of articles (%)**	**Article citation**
**Sample sources**		
Human clinical infection	65.3	(24–28, 36–39, 41–53, 56, 58, 60, 63, 64, 66, 72–75, 77, 78, 80–83, 86–92, 94, 96)
Human screening samples	52.8	(6, 20, 24, 25, 27, 42–48, 50, 52, 58, 60, 62–65, 72, 74–77, 81–83, 85, 87, 88, 90–96)
Retail meats	16.7	(11, 13, 25, 38, 39, 43, 55, 58, 63, 67, 69, 70)
Food animal samples	16.7	(8, 25, 38, 39, 43, 54, 58, 61, 63, 68, 71, 84)
Wastewater	11.1	(9, 38, 39, 41, 43, 58, 59, 63)
Hospital environment	11.1	(46, 51, 56, 60, 64, 87, 88, 94)
GenBank sequence	6.9	(11, 40, 57, 58, 90)
Milk products	4.2	(40, 43, 57)
Other	1.4	(65)
***Enterococcus*** **spp**.		
*E. faecium*	87.5	(6, 8, 9, 13, 20, 24–28, 36–39, 41–43, 45–52, 54–57, 59, 60, 62–67, 70–83, 85–96)
*E. faecalis*	34.7	(6, 8, 9, 13, 25, 26, 28, 36, 38, 40, 43, 44, 48, 53, 54, 58, 59, 61, 65, 66, 68, 69, 71, 78, 79)
*E. gallinarum*	13.9	(6, 8, 9, 13, 36, 38, 54, 59, 60, 67)
*E. hirae*	11.1	(8, 11, 13, 36, 38, 54, 59, 65)
*E durans*	9.7	(8, 13, 38, 43, 54, 59, 67)
*E. casseliflavus*	9.7	(6, 8, 9, 36, 38, 65, 81)
Other	13.9	(8, 11, 13, 36, 38, 43, 59, 65, 81, 84)
**VREfm specific study?**		
Yes	48.6	(20, 24, 27, 28, 37, 41, 42, 45–47, 50–52, 55, 62, 70, 72–77, 80, 82, 83, 85, 87, 88, 90–96)
No	51.4	(6, 8, 9, 11, 13, 25, 26, 36, 38–40, 43, 44, 48, 49, 53, 54, 56–61, 63–69, 71, 78, 79, 81, 84, 86, 89)

At least 14 different species of *Enterococcus* were described in the 72 articles (one article did not identify all species). The majority of articles (87.5%) described *Enterococcus faecium* with 35 (48.6%) of these articles specifically studying VREfm. Twenty-five articles (34.7%) identified *E. faecalis*, 10 articles (13.9%) *E. gallinarum*, eight articles (11.1%) *E. hirae*, and seven studies (9.7%) each described *E. casseliflavus* and *E. durans*. Ten articles described other species, including *E. villorum, E. thailandicus, E. cecorum, E. mundtii, E. pseudoavium, E. ratti, E. avium*, and *E. raffinosus*.

**Figure 3:**
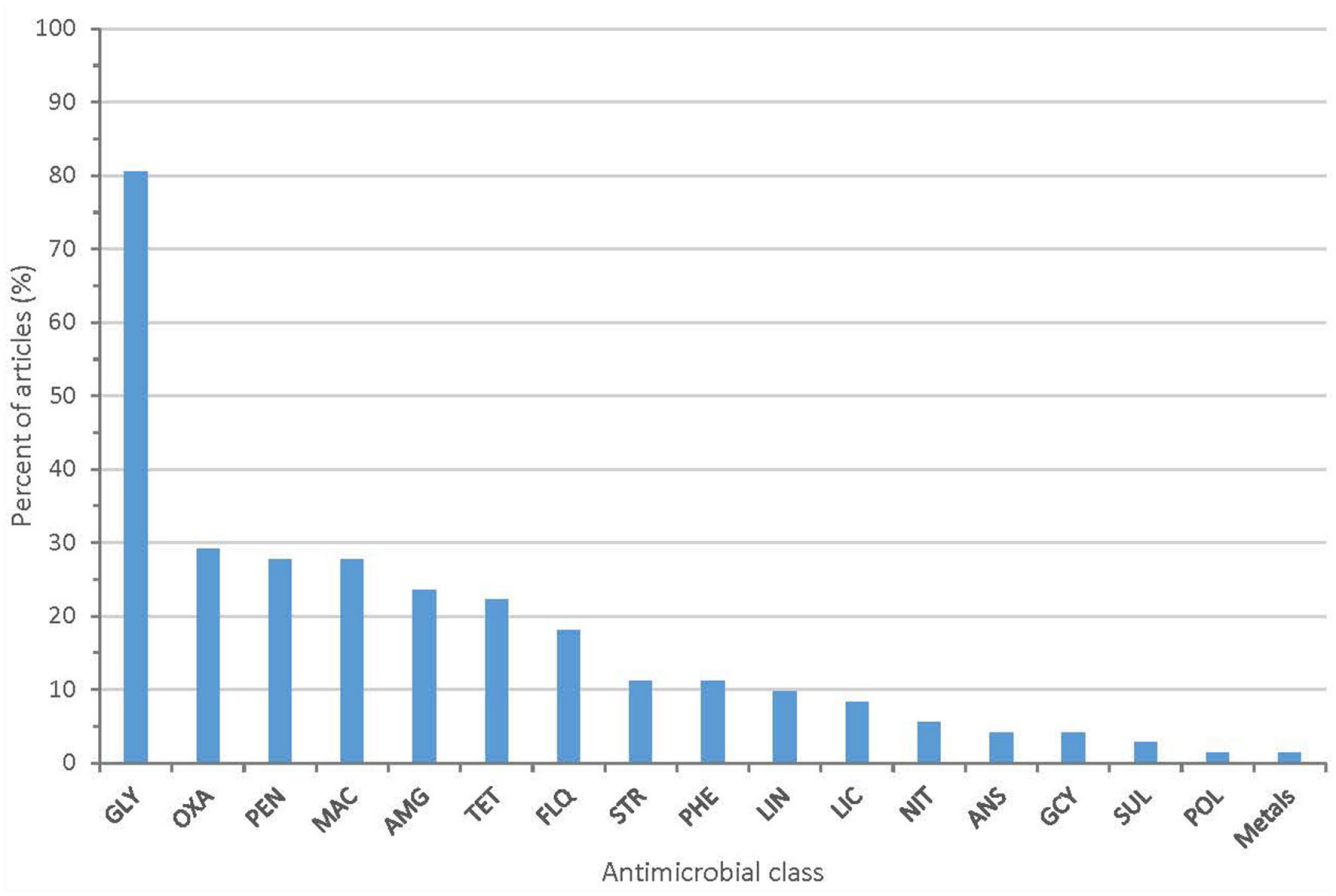
Proportion of articles included in scoping review identifying resistance to each antimicrobial class (GLY, glycopeptides; PEN, penicillins; MAC, macrolides; AMG, aminoglycosides; OXA, oxazolidinones; TET, tetracyclines; FLQ, fluoroquinolones; PHE, phenicols; LIN, lincopeptides; STR, streptogramins; LIC, lincosamides; ANS, ansamycins; GCY, glycylcyclines; NIT, nitrofurans; SUL, sulfonamides; POL, polypeptides).

All 72 articles provided some information on the AMR phenotypes of their isolates. The majority of articles (80.6%) describe isolates with resistance to glycopeptide antibiotics (vancomycin or teicoplanin). Twenty-one articles (29.2%) described isolates with resistance to oxazolidinones (linezolid or tedizolid). Other antimicrobial classes with identified phenotypic resistance included fluoroquinolones, macrolides, aminoglycosides, penicillins, and tetracyclines (Figure 3 and Supplementary Table 3). Methods used to assess phenotypic resistance in each article are outlined in Supplementary Table 4. Nineteen articles (26.4%) provided no information on the methodology used to define phenotypic resistance.

### WGS Platforms and Results

All articles selected performed whole genome sequencing of the isolates (as a part of the inclusion criteria) and Illumina was the most commonly used platform for sequencing. Sixty-four articles (88.9%) used a version of Illumina for sequencing, whereas four articles did not provide sequencing methodology. Illumina MiSeq was the most commonly used version of Illumina employed. Other platforms included PacBio, Ion Torrent PGM, or Illumina in combination with PacBio, Ion Torrent PGM, or MinIon platforms (Table 6). Sixty-two articles (86.1%) provided archive accession numbers for access to their resultant sequences.

**Table 6:** Summary of WGS platforms used in each article included in scoping review and whether archive accession numbers were provided in the article.

**Sequencing platform**		**Proportion of articles (%)**	**Article citation**
**WGS platform**			
Illumina	MiSeq	48.6	(6, 8, 9, 24–28, 38, 40, 44, 46, 53, 57, 60, 61, 66, 68, 72, 73, 78–80, 82–88, 90, 92, 94–96)
	NextSeq	9.7	(20, 50–52, 54, 55, 80)
	HiSeq	11.1	(41, 43, 59, 63–65, 69, 89)
	Version not specified	6.9	(13, 49, 56, 67, 76)
Illumina &.	PacBio	6.9	(39, 47, 71, 74, 75)
	Ion Torrent	2.8	(42, 48)
	MinIon	4.2	(62, 70, 81)
PacBio		2.8	(11, 91)
IonTorrent PGM		2.8	(37, 45)
No information		5.6	(36, 58, 77, 93)
**Archive accession numbers provided?**			
Yes		86.1	(6, 8, 9, 11, 13, 24, 26, 27, 37–45, 47–66, 68–72, 74–86, 89–95)
No		13.9	(20, 25, 28, 36, 46, 67, 73, 87, 88, 96)

**Figure 4:**
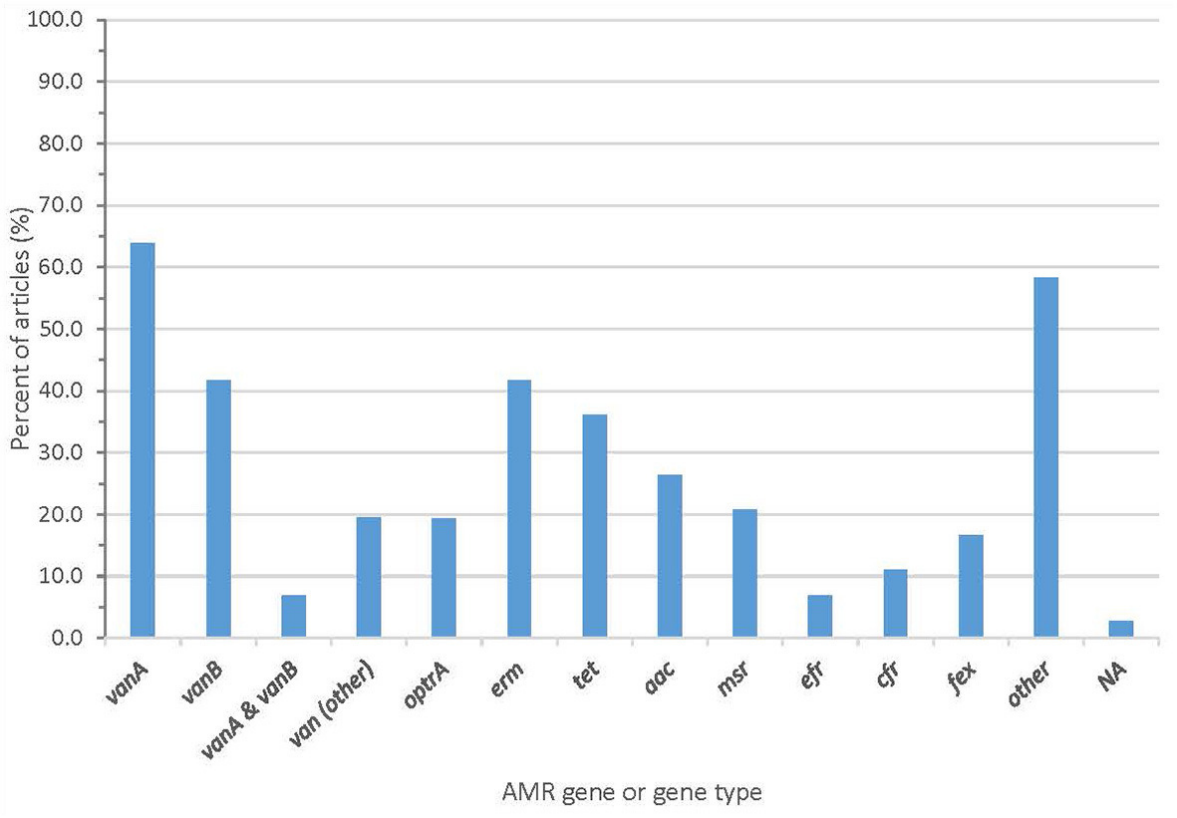
Proportion of articles included in scoping review finding an AMR gene or gene type.

Whole genome sequencing generated information about AMR genes that was described in almost all articles, with two articles (2.8%) failing to report AMR genes (Figure 4 and Supplementary Table 3). Forty-one articles (56.9%) reported the vanA gene cluster, 26 articles (36.1%) reported vanB, and 15 articles (20.8%) reported optrA.

**Figure 5:**
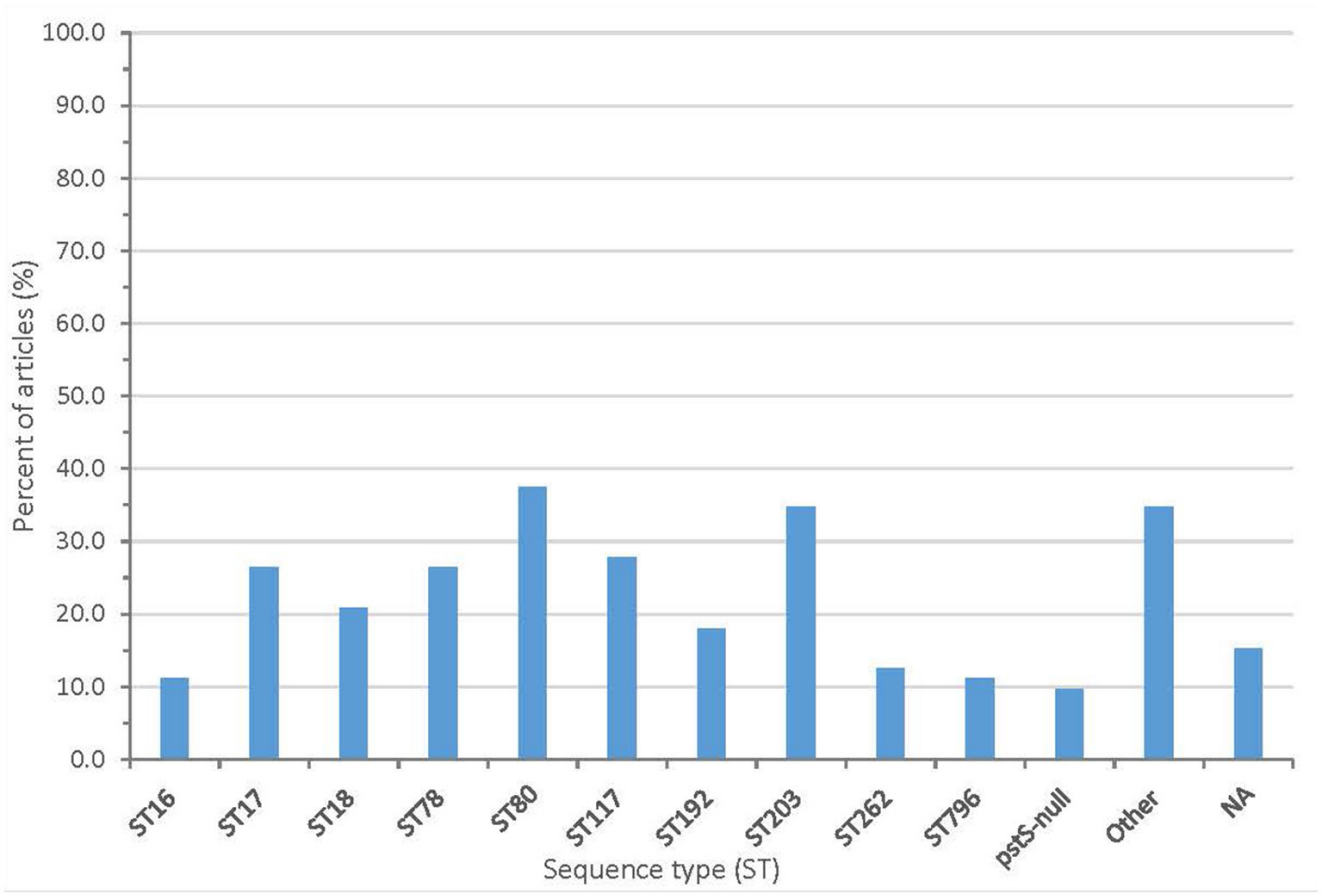
Proportion of articles included in scoping review finding different sequence types (NA, no sequence type described).

From the sequencing, 61 articles (84.7%) described the sequence type (ST) and/or clonal complex (CC) of isolates. Over 60 sequence types were reported, including ST203 (25 articles), ST80 (27 articles), ST78 (19 articles), and ST18 (15 articles). Seven articles reported a pstS-null isolate. The summary of clonal complexes is in Figure 5 and Supplementary Table 3.

Many articles (75.0%) reported other molecular techniques, including PCR, PFGE, and MALDI-TOF MS. These techniques were primarily used for speciation or screening for AMR genes (Supplementary Table 4).

### Article Findings and Conclusions

The articles' findings or conclusions related to antimicrobial resistance were summarized into categories, and articles could fit into more than one category. Of all articles, 31.9% reported a new or uncommon finding, which could include a novel strain or gene, or a previously described finding in a novel location. A fifth (20.8%) of articles reported how *Enterococcus* spp. were optimized for adaptation and survival in their environment. Twelve articles (16.7%) specifically stated that WGS was a better means of detecting or differentiating *Enterococcus* spp. than other genomic methods (such as PCR or PFGE). The other summarized findings are described in Table 7 with more details are available in Supplementary Table 2.

**Table 7:** Main categories of findings and conclusions and proportion of articles included in scoping review within each category.

**Finding and/or conclusion**	**Proportion of articles (%)**	**Article citation**
Report of a new or uncommon finding	31.9	(6, 28, 37, 43–45, 49, 52, 53, 59–63, 66, 69, 71, 74, 76, 77, 81, 86, 87)
*Enterococcus* is well optimized for adaptation and survival in their environment	20.8	(8, 9, 11, 26, 39, 46, 57, 62, 64, 68, 75, 78, 89, 94, 95)
WGS is the best means of detecting and differentiating *Enterococcus* spp.	16.7	(20, 27, 42, 50, 64, 72, 74, 80, 82, 88, 91, 92)
Surveillance of *Enterococcus* spp. is important	11.1	(28, 69, 73, 81, 82, 88–90)
Finding of suspected HGT or co-selection	9.7	(11, 49, 58, 59, 68, 70, 75)
Other^*^	16.7	(6, 38, 47, 48, 65, 78, 79, 83, 84, 91, 93, 95)
Importance of infection and control measures in hospitals	11.1	(20, 27, 41, 79, 87, 88, 94, 95)
VREfm prevalence is increasing in hospitals	11.1	(25, 28, 73, 74, 82, 85, 88, 96)
Human clinical strains have a higher number of ARGs than animal or human screening isolates or no relationship found between human/animal strains	9.7	(27, 38–40, 54, 57, 63)
Animal strains can carry ARGs and MGEs	8.3	(8, 13, 39, 41, 70, 84)
Information on antimicrobials and related resistance	8.3	(24, 44, 47, 53, 56, 67)
Finding of a shift to a new dominant strain	11.1	(50, 53, 72, 75, 80, 83, 85, 96)
Surveillance Program Report	5.6	(24, 25, 36, 51)
There is apparent transmission between human and animal enterococci (either human to animal or animal to human)	4.2	(43, 55, 58)
Importance of screening in hospitals	2.8	(72, 87)
Human wastewater contains human clinical strains	2.8	(9, 41)

* *Other refers to article findings that did not fit into another defined category*.

## Discussion

In this scoping review, we aimed to assess the value added by the use of WGS in the surveillance of enterococcal AMR. The use of WGS for the surveillance of *Enterococcus* spp. has added to our knowledge about *Enterococcus* spp. through the detection of previously unidentified strains and finding that WGS was better at detecting and differentiating *Enterococcus* spp. than other genomic methods. European countries provided the most surveillance studies, with many of these coming from the Danish DANMAP program. This is perhaps unsurprising as DANMAP is an extensive and well-established program implemented in 1995 (25).

### Importance of WGS to Detect AMR *Enterococcus* spp.

While the majority of *Enterococcus* spp. are adapted to the natural environment and animal GITs, rarely causing disease in humans, *E. faecium* and *E. faecalis* are the species most likely to cause human disease (2). Thus, it was not surprising that they were the most studied species in the included articles and the majority of articles were conducted in hospital settings. Nearly half of the included articles were studies specific to VREfm, showing the importance of vancomycin resistance in enterococci. WGS was important in studies to better understand VRE, especially in the assessment for vancomycin-variable enterococci (VVE). These are enterococcal isolates that are phenotypically sensitive to vancomycin but carry vancomycin-resistance genes. These isolates become phenotypically resistant to vancomycin when exposed to the antibiotic *in vivo* (25). Even though the resistance genes could be identified via PCR, the importance of WGS to further characterize VVE isolates was shown in the 2018 DANMAP report. The use of WGS and cgMLST allowed for the identification of new complexes and sequence types. DANMAP can now perform surveillance specific to these VVE strains. This should allow for earlier detection of VVE in patients and more appropriate treatment (25). WGS also allowed for a better understanding of *E. faecium* as it determined new sequence types, including *pstS*-null types, through cgMLST (37). The *pstS* gene locus is a housekeeping gene used for MLST but is missing in some VREfm strains. The use of WGS and cgMLST allowed for more robust sequence typing and identification of these isolates (37). Surveillance of enterococci within a hospital using WGS also allowed for the identification of a VREfm outbreak. A combination of sequencing data and an epidemiological investigation allowed for the identification of transmission route and the implementation of measures to prevent further outbreaks (20).

### A One-Health Approach

The ubiquitous nature of *Enterococcus* spp. naturally requires a One-Health approach to the surveillance of AMR in enterococci (38). This means a transdisciplinary approach across the human-animal-environment continuum in order to better understand the problem of AMR in enterococci (97). While enterococcal species other than *E. faecium* and *E. faecalis* were discussed in a few studies, in general, there were relatively few studies using a One-Health approach to compare animal, environmental (e.g., water and soil), and human samples (9, 11, 13, 39–41, 68–71). No studies sampled companion animals or equids, even though these animals live in close proximity to humans. This could be due to the complexity and cost of coordinating a study with so many sample sources or that human health studies are more easily funded. A collaborative transdisciplinary approach would bring a broad perspective to study design and allow for a better interpretation of the results. This would ease the complexity of designing and coordinating a One-Health surveillance study and create a more robust understanding of the issue of AMR in enterococci. Two studies included in this review did produce very informative results across multiple sample sources to address the One-Health continuum (38, 39). These studies pulled samples from livestock, retail meat, wastewater, and human bloodstream infections and showed limited sharing of genes between isolates from humans and animals (38, 39). Research using this One-Health approach will provide a means to assess the risk of AMR enterococci moving from food animals, through the food chain, into human populations as well as through the environment.

### Importance of Surveillance of Enterococci

Many articles included in this study stressed the importance of the surveillance of *Enterococcus* spp., especially in hospital settings. This is because enterococci are optimized for adaptation and survival in their environment, whether the hospital environment or natural environment (42, 72). Both targeted surveillance of at-risk patients (e.g., immunocompromised) and passive surveillance of incoming hospital patients allowed for early recognition of outbreaks. Outbreaks could be controlled before becoming a significant problem and new hospital protocols surrounding cleaning and isolation could be developed (20, 72, 87). WGS allowed for more accurate sequence typing and identification of AMR genes (27, 73, 88).

### Sequencing Platforms

From this review, Illumina sequencing platforms are currently the most popular for whole genome sequencing studies. They are historically reliable, with low error rates and have become accessible, abundant, and cost-effective (98). The combination of Illumina short-read with long-read sequencing (usually PacBio) was occasionally used to close a chromosome or a plasmid to accomplish genomic integrity as well as complete understanding of MGEs and their context. Unfortunately, the cost of running large numbers of isolates on a PacBio system is prohibitive (99). The use of long-read sequencing is likely to increase as inexpensive and portable bench-top platforms such as the Nanopore MinION become more reliable with lower error rates (100, 101). This will allow for the rapid identification of an isolate and its genetic composition, including MGEs (99). The majority of papers (86.1%) also provided archive accession numbers for their sequences, highlighting the importance of sharing raw genomic data with the scientific community and the requirements for publication in many journals.

### Limitations

This scoping review held limitations similar to other review papers in the possible omission of relevant literature, such as gray literature or articles written in a language other than English. Findings from government surveillance programs may be published online or as peer-reviewed articles, but in order to maintain an efficient and reproducible search method, gray literature was only searched from the reference lists in the primary research articles included in the review. No separate gray literature search was performed, which may have resulted in the omission of relevant information. Two non-English articles were excluded in our search which otherwise might have been included.

In order to minimize the omission of articles, several databases were searched and inclusion criteria were intentionally left broad until the abstract screening steps. The authors did maintain a rigid definition of surveillance, which could have excluded epidemiological articles that did not fit the selection criteria. This largely eliminated studies on human clinical isolates of *Enterococcus* spp. as the isolates would have been selected for a study based on certain characteristics.

Another limitation of the study is that a critical appraisal of the included articles was not conducted. This was intentional as one of the objectives of the present study was to identify gaps in the literature, but it means that studies of lower and higher quality would carry equal weight. The findings of some studies may not share the same validity based on their study design, but this was not determined in this review.

## Conclusion and Future Directions

Whole genome sequencing has added value to the surveillance efforts of *Enterococcus* spp. by identifying new genes and strains, adding to the knowledge about its prevalence in various settings, and finding that WGS is a better means of detecting and differentiating *Enterococcus* spp. than other molecular methods. The ability of *Enterococcus* spp. to adapt and survive in its environment was frequently stated as a reason for the importance of using WGS for the surveillance of this bacterium. Future studies should focus on the state of *Enterococcus* spp. in companion animal veterinary medicine and determining the link between humans, animals, food products, and the environment for a better One-Health approach to *Enterococcus* spp. surveillance.

## Author Contributions

LR, KS, SLC, and SCC developed the research question and scoping review protocol. LR and KS performed the literature search, article screening, and data extraction of included articles. LR drafted the complete manuscript. SCC and SLC were secondary reviewers of articles and primary reviewers of the manuscript. All authors assisted with editing and content review of the manuscript.

## Conflict of Interest

The authors declare that the research was conducted in the absence of any commercial or financial relationships that could be construed as a potential conflict of interest.

## Footnotes

^1^https://www.ncbi.nlm.nih.gov/pubmed/

^2^https://apps.webofknowledge.com/WOS_GeneralSearch_input.do?product=WOS&search_mode=GeneralSearch&SID=5D5o7Naqz9xk9WXkIZ8&preferencesSaved=

^3^http://web.a.ebscohost.com.ezproxy.lib.ucalgary.ca/ehost/search/advanced?vid=0&sid=59def56a-2134-4464-bd34-368a168a49ab%40sessionmgr4008
