# Supplementary material for: The Role of Whole Genome Sequencing in the Surveillance of Antimicrobial Resistant Enterococcus spp.: A Scoping Review
Source: Front Public Health. 2021 Jun 10;9:599285. doi: 10.3389/fpubh.2021.599285 (PMC8222819; doi:10.3389/fpubh.2021.599285)
Supplement: Supplementary file 1 [file Data_Sheet_1.docx]

**Supplementary Material for the article: The Role of Whole Genome Sequencing in the Surveillance of Antimicrobial Resistant *Enterococcus* spp.: A Scoping Review**

Note: for all supplementary material, citations correspond to list in the main article.

**Supplementary Table S1. Country of corresponding author and surveillance group of included articles**

| **Author Country and Surveillance Group** | **Proportion of articles (%)** | **Article Citation** |
| --- | --- | --- |
| Country of corresponding author | | |
| Australia | 21.1 | (46, 49, 57-59, 62-64, 66, 80, 87, 89) |
| Brazil | 3.5 | (60, 67) |
| Canada | 7.0 | (8, 9, 37, 83) |
| China | 5.3 | (11, 42, 69) |
| Colombia | 1.8 | (95) |
| Denmark | 17.5 | (25, 27, 28, 39, 40, 43, 50, 56, 81, 82) |
| Germany | 5.3 | (48, 84, 85) |
| Italy | 1.8 | (61) |
| Japan | 1.8 | (88) |
| Netherlands | 7.0 | (6, 24, 47, 72) |
| Portugal | 3.5 | (55, 70) |
| Saudi Arabia | 1.8 | (65) |
| South Africa | 1.8 | (71) |
| South Korea | 1.8 | (96) |
| UK | 7.0 | (36, 41, 45, 94) |
| USA | 12.3 | (13, 20, 26, 44, 68, 86, 93) |
| Surveillance group | | |
| None listed | 49.1 | (8, 9, 11, 24, 36, 37, 41, 42, 45, 47, 55-72) |
| Government Funded  AGAR (Australia)  COIPARS (Columbia)  DANMAP (Denmark)  Japan Nosocomial Infections Surveillance (JANIS)  NARMS (USA)  PHAC (Canada)  Tasmania Infection Prevention and Control Healthcare Associated Infection Surveillance Program  MDRO Network Rhine-Main (Germany)  National Reference Centre for Staphylococci and Enterococci  US Center for Disease Control (CDC) | 3.5  1.8  14.0  1.8  1.8  1.8  3.5  1.8  1.8  1.8 | (80, 87)  (40)  (25, 27, 28, 39, 43, 50, 81, 82)  (88)  (44)  (83)  (46, 89)  (84)  (85)  (86) |
| Private/Industry Funded  SENTRY (JBI Laboratories)  USA Consumer’s Union | 3.5  1.8 | (26, 93)  (13) |
| Within hospital program  EDS-HAT (UPMC)  Monash Newborn Hospital Initiative  University Hospital Meunster  CUH Sepsis Screening Protocol  Other | 1.8  1.8  1.8  1.8  5.3 | (20)  (49)  (48)  (94)  (6, 95, 96) |

**Supplementary Table S2. Stated objectives and summarized findings and/or conclusions of included articles**

| Citation | Objectives (as quoted by cited paper) | Findings |
| --- | --- | --- |
| (6) | “To determine the prevalence of *vanA/B/C/D*-carrying enterococci, we designed a real-time PCR for *vanC1/2/3* and *vanD* and screened rectal swabs from 360 patients.” | Development of a rapid rtPCR for vanC/D if it is needed for screening  No relevant *vanC-* or *vanD-* positive enterococci found from screening within the hospital  Report of a rare ST17/CT154 vanD VRE from a clinical isolate |
| (8) | “In the current study, we selected twenty-one isolates of enterococci originating from bovine feces for whole-genome sequencing and comparative genomic analysis. We hypothesized that *E. faecium* and *E. faecalis* would present more genes coding for virulence and antibiotic resistance than other *Enterococcus* spp. isolate from bovine species.” | New insights into genetic differences among *Enterococcus* spp. isolates from bovids  *Enterococci* spp. isolated from the bovine GIT contain ARGs and MGEs  Strains may be specifically adapted to their environment |
| (9) | “Comparative genomics of enterococci isolated from conventional activated sludge (CAS) and biological aerated filter (BAF) WWTPs was conducted.” | *E. faecium* CC17 is typically human clinical isolate and found in treated and untreated wastewater – suggests that most of *E. faecium* found in WWTPs is from humans  *E. faecalis* and *E. faecium* have smaller genomes than *E. gallinarum* or *E. casseliflavus* but have more virulence, AMR genes and MGEs – *E. gallinarum* & *E. casseliflavus* are better adapted to environment, *E. faecalis* & *E. faecium* are more virulent |
| (11) | “to describe the genome of a daptomycin-resistant *E. hirae* R17 recovered from a food sample”  “to investigate the genetic basis underpinning its antimicrobial resistance phenotype, virulence, and environmental adaptability” | Two possible virulence factors were found - likely acquired by HGT & virulence factors were similar to hospital strain *E. faecium* & *E. faecalis*  Contained lsa gene (clindamycin resistance); other ARGs likely also acquired by HGT - *E. hirae* R17 has strong genomic plasticity  First report of DAP-resistant *E. hirae*  Potential resistance to multiple antibacterial biocides indicates an ability to withstand adverse environments |
| (13) | “Using genomic and molecular tools, as well as additional antibiotic susceptibility testing, we further characterized the Consumers Union collection of chicken-meat associated enterococci (CUE collection) to investigate their drug resistance and genetic relationships, including phylogenetic and gene-based comparisons with known clinical isolates.” | Enterococci in poultry meat from poultry RWA have fewer ARGs than conventional  There were many MDR isolates from this study, including to human drugs  *E. gallinarum* only isolate with *vanC* gene (only VRE found) |
| (20) | “Following the initiation of WGS surveillance in our hospital, we identified a cluster of infections caused by vancomycin-resistance *E. faecium* strains that were genetically highly related, suggesting a common route of transmission. We conducted an epidemiological investigation to identify the route of transmission and implemented interventions to prevent further infections.” | Successful recognition and investigation of a previously unrecognized VREfm outbreak through WGS surveillance in a hospital (proof of concept for use of WGS surveillance to detect outbreaks and identify a transmission route)  Application of interventions |
| (24) | “to perform a point prevalence study on ESBL/plasmid mediated AmpC (pAmpC)/CP-producing *Enterobacteriaceae* and HA *E. faecium* (VRE and ARE) in hospitals in the Northern Dutch-German border region and to determine the predominant resistance genes.” | Reports the molecular epidemiology of HA VRE & ARE in Northern-Dutch-German border region  ARE & VRE carriage were associated with prolonged hospitalization and antibiotic use |
| (25) | “The key objectives of DANMAP are:  • To establish the "state of the nation" on to the use of antimicrobial agents in food animals and humans  • To collect and collate available national data on the occurrence of antimicrobial resistance in bacteria isolated from food animals, food of animal origin (e.g. meat) and humans.  • To identify areas for further research e.g. in transmission of resistance or possible associations between antimicrobial consumption and antimicrobial resistance  • To ensure data availability for veterinarians, medical doctors and other health professionals for the development of antibiotic guidelines for treatment  • To act as a knowledge base for authorities and politicians when performing risk assessment and management, thus supporting decision making in the prevention and control of resistant bacterial infections” | Increase in invasive *E. faecium* infection has led to an overall increase in invasive enterococci infections  Increase in VVE/VRE concerning, especially VVE clone ST1421-CT1134 as it is underdiagnosed  LVREfm were also detected (no LVREfs)  Summarises the results of susceptibility testing of isolates obtained from hospitals, general practice, veterinary practice, food-industry laboratories and the Danish Veterinary and Food Administration; as well as records of the types and amounts of antimicrobials prescribed for animal and human treatment in Denmark during 2018. |
| (26) | “[to evaluate] the oxazolidinone resistance mechanisms among a global collection of enterococcal clinical isolates. The epidemiology of *optrA*-carrying isolates and the *optrA* genetic context were determined.” | Prevalence of linezolid resistance is low  There are distinct resistance mechanisms between *E. faecalis* and *E. faecium*  *optrA* is a more prevalent resistance mechanism than 23S rRNA mutation for linezolid resistance  Global dissemination of *optrA* carrying *E. faecalis* (with diverse genetic background) |
| (27) | “to investigate the epidemiology and clonal relatedness of VREfm isolates in Danish hospitals from January 2012-April 2013.”  “to evaluate if WGS, in terms of ability to distinguish outbreaks, can replace PFGE for national surveillance of VREfm.” | VREfm is higher in clinical isolates than screening isolates  A polyclonal structure of VREfm outbreak in Denmark: 7 VREfm subgroups of VREfm  Direct or indirect transmission between patients is likely - infection & control measures in hospitals important against the spread of VREfm!  WGS is the most accurate typing method when compared to MLST and PFGE (preferred method in Denmark) |
| (28) | “to describe the occurrence of the *vanA* and *vanB* gene clusters among clinical *E. faecium* and *E. faecalis* in Denmark from 2005-2015 and to apply MLST and cgMLST to infer the genetic relatedness of *E. faecium* to understand the huge increase in *vanA E. faecium* in Denmark.” | Sub-clustering using cgMLST is useful for detecting emerging clones and outbreaks  The increase in *vanA E. faecium* followed a similar regional increase in nosocomial *C. difficile* (CD027) infections, usually treated with vancomycin – possible selection for *vanA E. faecium?*  A new type, ST203-CT859 emerged in Denmark in 2015 |
| (55) | “to thoroughly characterize the first (to the best of our knowledge) *optrA*-carrying strains obtained from an African country. The complete sequence of the plasmid pAF379, on which *optrA* was located, is also provided.” | First known report of *optrA* in *E. faecalis* in the African continent - the gene has now been found on all continents  Snapshot of gene diversity encoding resistance to non-fluorinated phenicols, fluorinated & non-fluorinated phenicols, and phenicols + oxazolidinones  Observed link between clinical and swine enterococci and healthy human and poultry enterococci  Sequence of another plasmid (pAF379) carrying *optrA* |
| (56) | “to characterise the genetic background of vancomycin-resistant *E. faecalis* (VREfs) in nine hospitals and performed WGS on all available VREfs detected in two departments of clinical microbiology in Copenhagen, Denmark, from January 2010 to February 2016.” | Only found *vanB* mediated vancomycin resistance in the VREfs isolates  14/15 VREfs isolates belonged to two hospital-adapted clones (ST6(CC2) & ST28(CC87)), whereas the VSEfs all belonged to a genetically different background |
| (57) | “To investigate the genetic context associated with the emergence of *vanA* VRE in Australia.” | Multiple STs in each city, except Sydney which only had ST80  Hospital-adapted clone, CC17, was the main clone from which *vanA* has emerged  Emergence of vanA in 2011-2013 unlikely to be a dissemination of a single ST or plasmid (given variety in plasmids)  Likely a multi-factorial emergence |
| (41) | “present the findings of the first large-scale whole-genome sequencing study of *E. faecium* from livestock and clinical isolates and the most comprehensive analysis to date of *E. faecium* from different reservoirs… first comparing the core genomes of *E. faecium* isolates from different reservoirs to determine phylogenetic relatedness and second, defining the extent to which genes and genetic elements in the accessory genome (including those encoding drug resistance) were shared.” | No livestock VREfm in this study  High relatedness between bloodstream infection isolates and wastewater isolates  Minimal meat VREfm - should cook out so low zoonotic risk  *vanA* plasmid in poultry VREfm from poultry lineage highly homologous to pVEF4 in Norwegian poultry (1998) and Danish chicken meat (2018) 🡪 widespread geographic and temporal dissemination  Host adapted populations of *E. faecium* |
| (42) | “a total of 78 genomes of *E. faecalis*… were subjected to comparative genomic analysis… the results from this comparative genomic analysis can provide the insight necessary to understand the genetic relationships between these *E. faecalis* strains and the adaptive mechanisms that have evolved to allow them to occupy different niches.” | Core genes encoding for ATP-binding cassette (ABC) transport system permease and ABC subfamily B made up most of the core genes for defense systems (confer MDR)  There were significantly more ARGs in strains from human blood sources than from those of dairy and water – indicates that the habitat contributes to the shaping of the genome |
| (58) | “To describe the transmission dynamics of the emergence and persistence of *vanA* vancomycin-resistant enterococcus (VRE) in an intensive care unit (ICU) using whole-genome sequencing of patient and environmental isolates.” | Environmental reservoirs were important in the spread of *vanA* VRE  Colonization was typically *vanB*  Nosocomial acquisition was predominantly *vanA* - characteristics for long term environmental survival? |
| (59) | “To explore further the potential reasons for the emerging dominance of *vanA*-VREfm in our region, we conducted a population-level study of VREfm… [to] provide an in depth assessment of the genomic diversity of *E. faecium* and, situating these in context with global VREfm, novel insights into the genomic epidemiology of this clinically relevant pathogen.” | Increase in *vanA* is not due to a single clone, but multiple introductions of *vanA* and that it is not all due to HGT, but also recombination events within the chromosome  Few putative virulence factors were found  Each *vanA* plasmid in the major STs was unique  Genetic diversity was lower across hospitals than within them - potential community reservoir? |
| (60) | “to characterize the lineage, virulence and resistance profile of VRE infection and colonization isolates in the context of their impact on the clinical outcome of haematology patients.” | In this hospital, *E. faecium* was the predominant VRE species and most STs belonged to CC17 (hospital acquired, virulent)  Virulence from VRE infection isolates was higher than in colonization isolates. |
| (61) | “to assess the presence of HA-*E. faecium* at the Hue Central Hospital, Vietnam, and to characterize isolates, including WGS of representative isolates… this is the first study describing clinical VREfm genotypes in Vietnam.” | First study with genetic characterization of Vietnamese nosocomial MDR *E. faecium*  Heterogenous population of HA-VSEfm/VREfm spread across hospital wards  Described lateral spread of *vanB2-Tn1549*-like transposon (teicoplanin resistance) among different HA-*E. faecium*  Finding of 2 *vanA/vanB2* type strains is unusual |
| (62) | “To characterize better the extent and significance of this lineage and its apparent spread to disparate geographical regions, we applied genome wide analysis to examine *vanA*-carrying *E. faecium* strains isolated from patients within ICU wards across the jurisdiction of New South Wales, Australia.” | Increase in *vanA* VRE prevalence throughout Australia  Confirmed presence of non-typeable ST17N and emergence of ST80N in Australia (pst-null)  Further supports use of WGS for the surveillance of Enterococcus |
| (63) | “[to investigate] the risk factors and origins of the first known occurrence of VRE colonization in the neonatal intensive care unit (NICU) at the Canberra Hospital.” | The outbreak in this NICU/SCN was in a tight cluster (ST1421) so likely only one clone (probably from an endemic ST1421 clonal lineage)  Only gestational age was significantly associated with colonization (after adjustment) |
| (64) | “to describe the emergence of *vanA* VREfm in the hematology/oncology and bone marrow transplant units, and in particular, the discovery of DNSEfm among this group. Whole genome sequencing (WGS) was performed to explore the relatedness of isolates in an attempt to better understand the emergence.” | First report of a DNSEfm cluster in Australia  Emergence of daptomycin resistance is likely due to underdosing of daptomycin |
| (65) | “to perform antibiotic susceptibility, and genomic analysis of clinical *E. faecalis* isolates from the western part of [Saudi Arabia]. The isolates were evaluated for the presence of virulence and antimicrobial resistance genes (ARGs). MLST analysis was performed to track the global distribution of the *E. faecalis* sequence types (STs) identified in the study.” | Linezolid, tigecycline & vancomycin had >95% activity; QD, clindamycin, erythromycin had almost no coverage; high dose streptomycin, gentamycin, and ciprofloxacin had suboptimal coverage  There is a diverse geographical distribution of MDR E. faecalis isolates in Saudi Arabia  VREfs is increasing and 17 distinct STs were found in this study  First study of the population structure of *E. faecalis* in Western Saudi Arabia  In Saudi Arabia, ampicillin, tigecycline, and linezolid are suggested as treatment options for combatting aminoglycoside- and macrolide- resistant *E. faecalis* |
| (45) | “describe a study that aimed to use wastewater to generate indirect evidence for the extent to which healthcare-associated *E. faecium* is disseminated in the community. This took an integrated approach that combined microbiological, epidemiological, and bacterial whole-genome sequence data and compared the genetic relatedness and presence of antibiotic resistance and putative virulence genes in *E. faecium* from sewage and patients with bloodstream infection in the same geographic region.” | The wastewater across the East of England has widespread VREfm from hospital-adapted lineages  Wastewater treatment doesn't necessarily prevent downstream environmental contamination  UV lights in last stage of treatment are effective against VREfm  Food chain VREfm seems to differ genetically from human and wastewater VREfm  Strong evidence for widespread dissemination of highly related healthcare associated drug resistant *E. faecium* lineages |
| (47) | “describe a retrospective WGS analysis of nosocomial VRE outbreak for which AFLP was performed as the routine typing method, and investigate the value of WGS for management of VRE outbreaks compared to AFLP.” | WGS has higher discriminatory power than AFLP  AFLP is as reliable for more genetically stable bacteria (i.e. not VRE)  Less than 7 core-SNPs is an appropriate cut-off for relatedness of epidemiologically linked VRE isolates (using WGS data in a hospital outbreak) |
| (36) | “use WGS to identify four VREfm isolates from two Scottish hospitals, which lack the *pstS* locus, and another Scottish VREfm isolate with a MLST profile, that to the best of our knowledge, has not been previously reported in the UK. Additionally, we provide information regarding their resistance profiles and epidemiology.” | New MLST profile for VREfm  Report of first non-typeable VREfm in Scotland  Phylogeny suggests inter-continental spread between Australia and Scotland  There is a strong association between *pstS*-null, *vanA*-type VREfm and an insertion in the *tetM* gene leading to decreased tetracycline resistance |
| (66) | “to investigate the antimicrobial resistance and genomic characteristics of *E. faecium* and *E. faecalis* isolated from the gut of Australian meat chickens at slaughter. Using whole-genome sequencing, we also investigated the evolution and genetic traits of a collection of Australian isolates of *E. faecium* obtained from cases of sepsis in humans to understand whether *E. faecium* originating in chicken was a possible cause.” | No clinical resistance to critically important human Abx (vancomycin, gentamicin) in Australian poultry; no *cfr* or *optrA* genes (which confer linezolid resistance)  High QD resistance possibly driven by use of virginiamycin  5 clades were found when poultry was compared to human isolates with minimal overlap  Poultry *E. faecium* is not a primary source of VREfm in Australian humans |
| (67) | “report for the first time the occurrence and genomic features of MDR vancomycin-resistant *Enterococcus faecium vanA* in marine brown mussels (*Perna perna*) from anthropogenically affected coastal waters of the Atlantic coast of Brazil.” | Filter feeder animals along the coast of Brazil could carry VRE leading to further dissemination  Likely caused by human contamination |
| (68) | “[To perform] WGS and comparative analysis on 41 newly sequenced isolates from the USA and 8 newly sequenced isolates from Pakistan.” | Linezolid resistance mechanisms differed between geographical locations (USA & Pakistan) |
| (69) | “To gain deeper insights into the genetic relationships of the dairy isolates and the isolates from human and animals, this study performed a comparative genomic analysis on 161 *E. faecium*, of which 54 dairy isolates were isolated and sequenced by our laboratory while the other 107 genome sequences were retrieved from a public genome database.” | The isolates from dairy and humans were genetically distinct  Dairy isolates were dispersed into several different areas of the tree and did not cluster on geographic proximity - likely did not arise from a common, recent ancestor  136/202 environment-specific genes found were dairy-specific  The ability of *E. faecium* to occupy broad ecological roles shows genomic plasticity and versatility |
| (37) | "The objectives of this research were to determine the extent to which beef cattle may contribute to AMR of indicator bacteria from feedlot and downstream environmental reservoirs relative to AMR in the public, and to characterize the potential chain of transmission to humans." | For the most part, human clinical and urban wastewater *E. faecium* and *E. faecalis* grouped into separate clades from cattle isolates  There is evidence for limited exchange of ARGs, virulence genes and plasmids between food animal and human isolates  A One-Health Study |
| (70) | "to address if *optrA*-carrying strains from different hosts carry similar genetic markers by performing a comprehensive comparison of publicly available *optrA*-carrying *E. faecalis* genomes." | Overall, there is a diverse population of optrA-positive *E. faecalis* with no apparent correlation between strain origin and phylogeny  There is a possible animal reservoir for linezolid resistant *E. faecalis* that could colonize humans  Proposition of a conserved chromosomal hotspot for the insertion of imbB-fexA-optrA plasmid  *E. faecalis* can share optrA-carrying platforms and plasmids with other Gram+ Firmicutes bacteria |
| (71) | "to elucidate the VRE resistance quotient (RQ) of different environmental matrices recovered from HW, influents (IW), activated sludge (AS), effluents of wastewater (EW), surface water (RW) and benthic sediments (RS) of water-receiving sewage effluents. In addition, a comparative genomic analysis of isolated strains recovered from the above environments was undertaken to highlight commonalities among the genetic elements associated with their resistomes... present in isolated VRE strains, to validate the hypothesis of spread through wastewater." | The first African study to compare VRE from hospital sewer systems to receiving water bodies which is important because of the use surface water by rural communities  VRE STs largely belonged to hospital-associated CC17, but no single ST followed the continuum from hospital wastewater to the aquatic environment  Evidence for MGE exchange with vancomycin resistance conferred by vanA, vanC, vanN, vanL, and vanG gene clusters |
| (72) | "to investigate the molecular characteristics and genetic relatedness of the first reported cases of vancomycin-resistant enterococci (VRE) from the Tripoli Medical Center, Libya" | First in-depth analysis of VRE in Libya and North Africa  The VRE in the Libyan health care system are genetically related to other European cladeA1 isolates (hospital-associated) |
| (80) | “to determine the proportion of *E. faecalis* and *E. faecium* bacteraemia isolates demonstrating antimicrobial resistance with particular emphasis on:   1. Assessing susceptibility to ampicillin 2. Assessing susceptibility to glycopeptides 3. Molecular epidemiology of *E. faecium*” | Insight into the *E. faecalis* and *E. faecium* populations causing clinical disease in humans in Australia  Notable conclusions:   - Prevalence of phenotypic VREfm bloodstream infections: 46.5% (192/413) - Prevalence of *vanA* and/or *vanB* genes in *E. faecium*: 49.3% (201/408) - Total number of *E. faecium* with van genes or phenotypic VRE: 203/413 (49.2%) |
| (40) | “This study aimed at characterizing the [COIPARS] isolates [from retail chicken collected in 2010-11] resistant to linezolid and detecting the resistance mechanism.” | First known report of *optrA* in *E. faecalis* in the Americas from retail poultry  Importance of surveillance and saving isolates and raw genomic data to analyze as new AMR genes are found |
| (39) | “present the genome sequences along with the main features of six *E. faecalis* strains showing the differences between Cu resistant and sensitive strains of *E. faecalis*, and suggesting possible co-transfer of Cu and antibiotic resistance determinants in these bacteria.” | Provide information regarding the co-transfer of genes encoding additional Cu resistance and genes encoding AMR  *tetM* was found in all Cu resistant strains - consistent with environmental MDR enterococcal strains |
| (50) | To investigate the increase in *vanA E. faecium* occurrence at Aarhus University Hospital, Central Denmark Region | Describes the emergence of *vanA* VRE at a Danish hospital in isolates obtained through surveillance |
| (81) | “The present study uses WGS to describe the genetic epidemiology of VREfm in the Capital Region of Denmark for an extended time period, 2012-2014, to assess the contribution of clonal spread as well as horizontal transfer of TN*1546*-like transposons in the dissemination of *vanA* VREfm. All Tn*1546*-like transposons were characterized to assess their similarity. One VREfm isolate was selected for PacBio long-read sequencing to close a *vanA* plasmid and to study the dissemination of this resistance plasmid.” | Increase in VREfm from 2012 onwards - partially due to increased screening and partially due to increased clinical cases  There is spread within wards, between wards, and between hospitals (patient transfer)  PacBio (long read) sequencing allowed closure of a *vanA* plasmid  Presence of a successful *vanA* carrying plasmid, pHvH-V24 (type 4 transposon) that has spread easily across several populations of *E. faecium*  Use of WGS allowed for greater discrimination of isolates, identifying clonal spread of VREfm between wards and hospitals in the Capital Region of Denmark |
| (43) | “to screen Danish chicken meat for VREfm using a selective enrichment and to genetically characterize the strains and plasmids obtained.” | Plasmid pVEF4_A is likely of poultry-origin and confers vancomycin resistance  This plasmid has persisted in poultry for many years with no apparent selective pressure (avoparcin banned for use in food production animals)  This plasmid is transferrable from carrier to a vancomycin susceptible *E. faecium* |
| (82) | “we applied short- and long-read sequencing along with epidemiological data to understand the sudden increase in VREfm in the low-prevalence Capital Region of Denmark from 2012 to 2015. The aims of this study were: (i) to analyze the genetic relatedness between temporally and geographically matched VREfm and VSEfm and to detect the spread of the vanA plasmid between different clones; (ii) to identify VREfm transmission events between hospitals; and (iii) to characterize the pan-genome and plasmids within the largest clonal group accounting for 40% of the VREfm isolates.” | Clonal and plasmid spread contributed to the rapid increase and persistence of *vanA* VREfm to a previously low-prevalence region  This region has a highly interlinked healthcare network which could contribute to the rapid transmission  There is no single optimal combination of accessory genes - VREfm isolates from this clone can rapidly acquire genes to optimize survival |
| (44) | “describe the first identification of plasmid-mediated linezolid resistance in bacteria from food animal caecal contents in the USA.” | One of the first reports of *optrA* in the USA, first report of *optrA* & *cfr* on the same plasmid in the USA (phenicol resistance)  Found genetically diverse linezolid resistant strains from different sources |
| (83) | “[to genetically characterize] a genomic island harbouring a new *vanD* allele from *Enterococcus faecium* N15-508.” | First complete genetic characterization of a novel strain type and *vanD* operon that has the potential to be mobile |
| (46) | “performed next-generation sequencing of VREfm isolates collected at the Royal Hobart Hospital (RHH)…We then correlated genomic information with epidemiological data to better define the hospital spread of VREfm.” | Shift in VREfm profile at RHH – VREfm now endemic; ST796 and ST80 now major; with *vanA* locus increasing in prevalence  Demonstrates importance of screening to detect isolates that may be involved in transmission  WGS helpful for more accurate typing of *E. faecium* isolates for better investigations  VREfm genotypes correlated with spatio-temporal patient movements which allowed for detection of potential points of transmission |
| (84) | “report on the genome-based analysis and comparison of VREfm isolated from patients with or without a prior history of hospitalization during admission to intensive care units or other wards with patients at risk for VREfm colonization/infection”  (region = Germany) | WGS-based analysis revealed the circulation of a new and near-ubiquitous VREfm clone (ST117/CT71/*vanB*) in and around Frankfurt am Main |
| (85) | “In order to investigate whether *optrA* is already circulating in German *Enterococcus* spp. clinical isolates and, moreover to assess the prevalence of the resistance locus amongst the high number of LRE received by the German NRC, the authors analysed the entire LRE strain collection retrospectively from 2007 until 2017 with respect to the presence of *optrA* variants thereof, adjacent genetic loci and transferability of the resistance locus.” | *optrA* is complex and can be integrated into a variety of plasmid backgrounds |
| (86) | "to improve our understanding of regional ARO dissemination by examining the distribution of ARO lineages across healthcare facilities and characterizing patient factors and prior healthcare exposures that are associated with the risk of colonization at NF admission." (focused on MRSA, VREfc, VREfm, CipREc) | Patient characteristics (physical disabilities and exposure to antibiotics), rather than specific transfer facility were better predictors of colonization with an antibiotic resistant organism  Colonization species are endemic to the healthcare facilities – regional antibiotic stewardship will be important to decrease the incidence of colonization |
| (87) | "To understand the high prevalence of nosocomial VREfm causing enterococcal bacteraemia in Australia, a whole genome sequencing (WGS) bioinformatics approach was used to determine the relationship and characteristics of *E. faecium* isolates from the 2015, 2016 and 2017 AESOPs." | WGS is better for surveillance versus eBURST phylogenetic analysis as it gives higher resolution and better identifies strains  The epidemiology of *E. faecium* is heterogenous across Australia, but in general vanA VREfm is increasing and vanB VREfm is decreasing in prevalence |
| (88) | To describe and attempt to identify the plasmid for the VanA-type VRE spread of a recently discovered transferable linear plasmid harboring vanA and vanM gene clusters from Efm in Japan | “first report of a local spread of VRE as a result of the transmission of a linear plasmid” (pELF2)  Conjugation experiments showed transfer of pELF2 to *E. faecium, E. faecalis, E. hirae,* and *E. casseliflavus* – shows the importance of surveillance of non- Efs or Efm |
| (89) | "In this work, we examined VREfm on a state-wide basis to improve our understanding of this pathogen across Tasmania. We determined the genotypes of VREfm isolates collected at Tasmania's other public hospitals… using multi-locus sequence typing (MLST), core genome MLST (cgMLST), and single-nucleotide polymorphic (SNP) analysis. We then combined genomic data with patient spatio-temporal information which provided insights into the emergence and distribution of VREfm sequence types in the state." | WGS-based surveillance is important to follow newly emerged and emerging VREfm STs in Tasmania  ST796 establishes quickly and out-competes other hospital strains of VREfm  There is a marked increase of VREfm in Tasmania  VREfm may be harboured in infected/colonized patients for several years |
| (93) | “report the characterization and genetic context of this *cfr* variant recently reported in *P. difficile* that was detected in two human clinical isolates of *Enterococcus faecium* from a medical center in New Orleans.” | New *cfr* plasmid gene discovered in *E. faecium* - first *cfr* in clinical isolates in USA & a new cfr variant (*cfr(B)*)  cfr(B) – found in MGEs and at least 3 bacterial species (*E. faecium*, *E. faecalis*, & *P. dificile*) |
| (49) | “describe an extensive outbreak of colonization with a newly emergent strain of VREfm [within a NICU] that was promptly terminated with the institution of contact precautions and enhanced environment cleaning.” | Description of *vanB* ST796 VREfm colonization outbreak in a neonatal service (few clinical isolates may represent a large silent outbreak of VREfm)  Possibility of environmental contamination as a colonization source  Importance of active surveillance to detect VREfm colonization  WGS and MLST are valuable to determine the strain type so that appropriate infection control can be implemented |
| (48) | “[To] investigate the impact of weekly VRE screening within the bundle of infection control measures to terminate VRE outbreaks on an oncologic ward.” | Weekly screening in addition to other infection control strategies can decrease nosocomial infection rates of VRE  This outbreak was predominately ST 192 & 203 (most common in German outbreaks) which is different from Denmark and the Netherlands which typically harbor ST 117 & 80 |
| (94) | “Here, we extend these findings [of a previous study] through the study of four patients who developed *E. faecium* bloodstream infection while undergoing longitudinal surveillance of stool carriage, and from whom multiple colonies were sequenced from stool, blood cultures, and their environment.” | Opportunistic infection with transition from carriage to invasive state is not associated with bacterial adaptation (such as with MRSA)  The stool and environment of a single patient likely share highly related isolates |
| (95) | "Here, we sought to characterize the population structure of VREfm lineages in a collection of isolates recovered between 1998-2015 in prospective multicentre studies performed in selected Latin-American hospitals. Using the Latin American genomes, we revisit the global population structure and evolutionary history of VREfm." | It is important for Latin America to implement more of their own surveillance programs for MDR organisms as the Latin American isolates seem to call into their own distinct population of clinically related isolates when compared to the global isolates. |
| (96) | "We have identified the nationwide emergence of VREF isolates lacking the *pstS* gene in Korea and hereby report the molecular characteristics of these isolates." | WGS is important for epidemiological study of nosocomial *E. faecium* infections  There is likely sporadic pstS gene deletion across South Korea and the first pstS-null isolate probably appeared in South Korea around 2006 |

**Supplementary Table S3. AMR phenotypes, resistance genes, and strain types (ST) and clonal complexes (CC) identified in included articles**

| **Finding** | **Proportion of articles (%)** | **Article citation** |
| --- | --- | --- |
| AMR Phenotypes (by antimicrobial class) | | |
| Glycopeptides (vancomycin, teicoplanin) | 84.2 | (6, 8, 9, 13, 20, 24, 25, 27, 28, 36, 37, 41, 43, 45-50, 56-68, 71, 72, 80-89, 93-96) |
| Beta-lactams (ampicillin, penicillin, carbapenem) | 29.8 | (9, 24, 25, 36, 37, 41, 45, 49, 61, 65-67, 72, 80, 82, 84, 95) |
| Macrolides (erythromycin, tylosin) | 28.1 | (8, 9, 11, 13, 25, 37, 40, 44, 55, 65-67, 72, 80, 88, 93) |
| Aminoglycosides (gentamicin, streptomycin, kanamycin, spectinomycin) | 28.1 | (8, 25, 36, 37, 40, 44, 49, 55, 61, 65, 66, 72, 80, 93-95) |
| Oxazolidinones (linezolid, tedizolid) | 26.3 | (13, 25, 26, 37, 40, 44, 60, 65, 66, 68, 70, 72, 80, 85, 93) |
| Tetracyclines (doxycycline, oxytetracycline, minocycline) | 22.8 | (8, 11, 13, 25, 36, 37, 40, 55, 65, 66, 72, 80, 93) |
| Fluoroquinolones (levofloxacin, ciprofloxacin) | 19.3 | (11, 25, 36, 40, 65, 67, 72, 80, 84, 93, 95) |
| Phenicols (chloramphenicol) | 8.8 | (13, 25, 40, 44, 55) |
| Lincopepetides (daptomycin) | 8.8 | (11, 13, 64, 68, 80) |
| Streptogramins (QD, virginiamycin) | 10.5 | (25, 37, 65, 66, 72, 94) |
| Lincosamides (lincomycin, clindamycin) | 7.0 | (13, 65, 66, 72) |
| Rifampicin | 5.3 | (36, 43, 67) |
| Glycylcyclines (tigecycline) | 5.3 | (13, 25, 37) |
| Nitrofurans (nitrofurantoin) | 7.0 | (36, 37, 72, 80) |
| TMS | 3.5 | (36, 72) |
| Polypeptides (bacitracin) | 1.8 | (11) |
| Metals (copper) | 1.8 | (39) |
| AMR genes | | |
| vanA | 68.5 | (6, 9, 24, 25, 27, 28, 36, 37, 39, 41-43, 45, 46, 48, 50, 57-65, 67, 69, 72, 80-82, 84, 85, 87-89, 93, 95, 96) |
| vanB | 42.1 | (6, 24, 25, 27, 28, 41, 42, 46-49, 56, 58, 59, 61, 62, 65, 69, 72, 80, 84, 87, 89, 95) |
| vanA + vanB | 7.0 | (48, 59, 80, 89) |
| Other van | 19.3 | (6, 8, 9, 13, 36, 67, 71, 72, 83, 88, 96) |
| optrA | 21.1 | (25, 26, 37, 40, 41, 44, 55, 65, 68, 70, 85, 95) |
| Erm | 38.6 | (8, 9, 36, 37, 40-42, 44, 55, 65-67, 70-72, 81, 82, 87, 88, 93, 95, 96) |
| Tet | 31.6 | (8, 9, 36, 37, 39-42, 45, 55, 66, 70-72, 82, 87, 93, 95) |
| Aac | 22.8 | (8, 9, 36, 37, 40, 41, 55, 69-71, 87, 93, 95) |
| Msr | 15.8 | (8, 9, 41, 66, 67, 69, 72, 82, 87) |
| Efr | 8.8 | (9, 36, 41, 42, 65) |
| Cfr | 10.5 | (25, 26, 68, 85, 93, 95) |
| Fex | 14.0 | (37, 40, 41, 44, 55, 68, 70, 95) |
| Other | 54.4 | (8, 9, 11, 24, 25, 36, 37, 39-45, 55, 60, 65-72, 81, 82, 87, 88, 94-96) |
| NA | 3.5 | (20, 86) |
| ST and/or CC | | |
| ST16 | 14.0 | (9, 37, 56, 65, 66, 70, 71, 85) |
| ST17 | 21.1 | (6, 24, 37, 45, 57, 59, 61, 80, 86, 87, 89, 95) |
| ST18 | 21.1 | (9, 27, 28, 45, 47, 50, 57, 61, 81, 86, 87, 95) |
| ST78 | 22.8 | (27, 45, 46, 57, 60, 61, 72, 80, 81, 85, 87, 89, 94) |
| ST80 | 33.3 | (25, 27, 28, 45-47, 57, 59, 71, 72, 80-82, 84-87, 89, 94) |
| ST117 | 17.5 | (25, 27, 28, 37, 45, 47, 50, 81, 82, 84) |
| ST192 | 15.8 | (27, 28, 46, 48, 50, 57, 82, 84, 87) |
| ST203 | 33.3 | (24, 25, 27, 28, 45-48, 50, 57, 59, 64, 71, 80-82, 86, 87, 89) |
| ST262 | 8.8 | (61, 80, 84, 87, 94) |
| ST796 | 14.0 | (46, 49, 57, 59, 64, 80, 87, 89) |
| pstS-null | 12.3 | (36, 59, 62, 80, 87, 89, 96) |
| Other | 61.4 | (8, 9, 20, 25, 27, 28, 37, 40, 43, 45-47, 50, 55-57, 59-61, 63, 65-67, 70, 71, 80, 83-87, 89, 93-95) |
| Clonal Complexes described | 15.8 | (9, 13, 25, 26, 60, 61, 82, 87, 94) |
| NA | 15.8 | (11, 39, 41, 42, 44, 58, 68, 69, 88) |

**Supplementary Table S4. Antimicrobial susceptibility testing method and other molecular methods performed in included articles**

|  | **Method** | **Percent of articles (%)** | **Citation** |
| --- | --- | --- | --- |
| Antimicrobial susceptibility testing method | VITEK2 | 24.6 | (6, 24, 36, 41, 45, 47-49, 63-65, 72, 94, 96) |
|  | Broth microdilution | 17.5 | (11, 13, 26, 36, 40, 44, 55, 60, 66, 67, 85, 88, 93, 95) |
|  | Etest | 14.0 | (6, 46-48, 55, 61, 64, 68) |
|  | Disc diffusion | 17.5 | (9, 25, 37, 41, 46, 61, 67, 68, 82, 89) |
|  | AM plates | 8.8 | (6, 58, 67, 71, 84) |
|  | Other | 5.3 | (61, 68, 84) |
|  | Sensititre | 1.8 | (43) |
|  | Not described | 31.6 | (8, 20, 27, 28, 39, 42, 50, 56, 57, 59, 62, 69, 70, 80, 81, 83, 86, 87) |
| Other molecular methods completed | PCR | 57.9 | (6, 13, 24-26, 28, 36, 37, 39, 40, 43, 46, 49, 50, 55, 57, 58, 60-65, 67, 72, 80, 83, 85, 86, 88, 93, 95, 96) |
|  | MALDI-TOF MS | 28.1 | (6, 24, 43, 45, 48, 49, 58, 63-66, 71, 72, 82, 89, 94) |
|  | PFGE | 17.5 | (8, 26, 27, 50, 60, 65, 85, 88, 93, 96) |
|  | MLST | 10.5 | (25, 27, 55, 61, 83, 96) |
|  | Other | 10.5 | (11, 37, 44, 47, 55, 93) |
|  | Not described | 21.1 | (9, 20, 41, 42, 56, 59, 68-70, 81, 84, 87) |
